# Restoring Metabolic-Inflammatory Homeostasis: Curcumin’s Multi-Layered Defense Against Chondrocyte Dysfunction

**DOI:** 10.3390/metabo16070506

**Published:** 2026-07-19

**Authors:** Cong Wang, Yanran Li, Huihui Meng, Gaocheng Shi, Yifan Sun, Ke Che, Hao Yu

**Affiliations:** School of Chinese Medicine, Bozhou University, Bozhou 236800, China; 2026070001@bzuu.edu.cn (C.W.);

**Keywords:** curcumin, osteoarthritis, metabolomics, machine learning

## Abstract

Background: Osteoarthritis (OA) pathogenesis involves inflammatory-metabolic crosstalk driving cartilage destruction, yet the mechanisms of potential therapeutics like curcumin remain poorly defined. Methods: We integrated untargeted metabolomics, transcriptomic analysis of four GEO datasets (GSE12021, GSE55235, GSE55457, GSE82107), and three machine learning algorithms (LASSO, Random Forest, XGBoost) to characterize curcumin’s effects on IL-1β-induced human chondrocytes. Results: Metabolomic profiling demonstrated that IL-1β caused significant depletion of TCA cycle intermediates compared to blank controls, including pyruvate (log2FC = −1.34, *p* < 0.001) and malate (log2FC = −0.54, *p* < 0.001). High-dose curcumin (10 μM) significantly restored these metabolites towards normal levels (pyruvate log2FC = −0.01 vs. model; malate log2FC = −0.20 vs. model). Three machine learning algorithms converged on a six-gene inflammatory-metabolic signature (*NFKBIA*, *MMP9*, *LCK*, *TDO2*, *HADHA*, *VEGFA*), all showing excellent discriminative performance for OA (individual AUCs > 0.75). qRT-PCR validation confirmed that high-dose curcumin significantly downregulated pro-inflammatory genes compared to IL-1β treatment alone: *JUN* (log2FC = −0.68, *p* < 0.001), *IL6* (log2FC = −1.13, *p* < 0.001), *PTGS2* (log2FC = −0.94, *p* < 0.001), *CCL20* (log2FC = −1.51, *p* < 0.001), and *MMP9* (log2FC = −0.42, *p* < 0.001). Conversely, curcumin significantly upregulated the NF-κB inhibitor *NFKBIA* (log2FC = 0.40, *p* < 0.001), whose expression was initially suppressed by IL-1β (−log2FC = 0.76 vs. blank, *p* < 0.001). Conclusions: This systems-level analysis suggests curcumin modulates metabolic-inflammatory networks in OA chondrocytes, with NFKBIA as a candidate mediator, offering a mechanistic framework for drug-like molecule development despite curcumin’s own translational limitations.

## 1. Introduction

Degenerative joint diseases affect over 500 million individuals worldwide, with osteoarthritis (OA) representing the most prevalent musculoskeletal disorder and a leading cause of disability among aging populations [1,2]. Despite the substantial global health burden, current pharmacological interventions remain limited to symptomatic management—primarily analgesics and non-steroidal anti-inflammatory drugs (NSAIDs)—with no approved disease-modifying OA drugs (DMOADs) capable of halting or reversing cartilage degradation [3,4]. The translational gap between mechanistic discoveries and effective therapies reflects, in part, the multifactorial nature of OA pathogenesis, which involves intricate interactions among mechanical stress, inflammatory cascades, metabolic dysfunction, and extracellular matrix degradation.

Recognition that inflammation and metabolism constitute interconnected regulatory networks has catalyzed a shift in OA research toward systems-level disease understanding [5,6]. During OA progression, articular chondrocytes undergo profound metabolic reprogramming characterized by a shift from mitochondrial oxidative phosphorylation toward aerobic glycolysis—a phenomenon reminiscent of the Warburg effect in cancer biology [7]. Pro-inflammatory cytokines, particularly IL-1β and TNF-α, drive this metabolic rewiring while simultaneously activating catabolic programs that degrade cartilage extracellular matrix [8]. This establishes a self-perpetuating cycle wherein metabolic dysfunction sustains inflammation, and inflammation further disrupts cellular bioenergetics [9,10]. Interrupting this vicious cycle requires interventions capable of simultaneously targeting multiple cellular processes, where traditional reductionist approaches often fail to capture biological complexity.

Curcumin, the bioactive polyphenolic compound from Curcuma longa (turmeric), has been extensively investigated as a potential OA therapeutic based on a substantial body of preclinical evidence demonstrating anti-inflammatory, antioxidant, and chondroprotective properties [11,12]. However, it is critical to acknowledge that curcumin is classified as a Pan-Assay Interference Compound (PAINS) and possesses significant pharmacological liabilities, including poor aqueous solubility, low oral bioavailability, rapid metabolic degradation, chemical instability under physiological conditions, and multiple modes of assay interference [13,14]. Furthermore, despite over 120 clinical trials investigating curcuminoids across various disease indications, no double-blind, placebo-controlled clinical trial has unequivocally demonstrated clinical efficacy for curcumin in OA or other conditions [13,14]. Additionally, as noted by Aggarwal et al., turmeric contains hundreds of potentially bioactive compounds beyond curcumin, and “curcumin-free” turmeric preparations retain significant anti-inflammatory and anticancer activities, raising important questions about whether the observed bioactivity can be exclusively attributed to curcumin [15]. These considerations underscore the need for careful and cautious interpretation of mechanistic studies involving curcumin.

Despite these limitations, curcumin remains a useful mechanistic probe for understanding metabolic-inflammatory crosstalk. Its pleiotropic effects—whether mediated through specific molecular targets or, as its PAINS classification suggests, through polypharmacology involving multiple low-affinity interactions—provide a unique opportunity to map the interconnected molecular networks that link metabolic dysfunction with inflammatory signaling in chondrocytes. However, to date, no integrated multi-omics study has systematically characterized curcumin’s effects on the metabolic-inflammatory interface in chondrocytes, representing a significant knowledge gap in our mechanistic understanding.

Recent advances in systems biology, particularly the integration of metabolomics and transcriptomics with machine learning algorithms, provide unprecedented opportunities to decipher complex pharmacological mechanisms at a network level [16,17]. Untargeted metabolomics captures the terminal metabolic phenotype reflecting the integrated output of genomic, transcriptomic, and proteomic regulation [18], while transcriptomic profiling reveals gene expression programs underlying disease states [19]. Machine learning algorithms can distill high-dimensional multi-omics data into core molecular signatures, enabling identification of key regulatory nodes [20,21]. However, whether curcumin’s anti-inflammatory effects represent secondary consequences of metabolic restoration—or vice versa—remains unresolved.

To address these knowledge gaps, we employed an integrated systems biology approach combining untargeted metabolomics, transcriptomic analysis, and machine learning to comprehensively characterize curcumin’s effects on IL-1β-induced chondrocyte dysfunction. Our integrative analysis reveals that curcumin is associated with coordinated restoration of TCA cycle intermediates and amino acid metabolism alongside modulation of a six-gene inflammatory-metabolic signature (NFKBIA, *MMP9*, *LCK*, *TDO2*, *HADHA,* *VEGFA*), with *NFKBIA* emerging as a promising candidate mediator of these responses. This study provides a systems-level perspective on metabolic-inflammatory network modulation and establishes a foundation for targeting metabolic-inflammatory networks in degenerative joint diseases, while maintaining appropriate scientific caution regarding curcumin’s pharmacological limitations.

## 2. Materials and Methods

### 2.1. Cell Culture and Treatment

Human articular chondrocytes were purchased from iCell Bioscience (Shanghai, China) and cultured in complete growth medium (DMEM/F12 supplemented with 10% fetal bovine serum, 100 U/mL penicillin, and 100 μg/mL streptomycin) at 37 °C in a humidified atmosphere containing 5% CO_2_. For all experiments, third-passage cells were seeded in 6-well plates at a density of approximately 1 × 10^6^ cells/well and cultured until reaching 80–90% confluence. Cells were then serum-starved for 12 h before treatment initiation.

Chondrocytes were randomly assigned to four experimental groups: (1) Blank (untreated control); (2) Model (10 ng/mL recombinant human IL-1β); (3) CurL (5 μM curcumin + 10 ng/mL IL-1β); and (4) CurH (10 μM curcumin + 10 ng/mL IL-1β). Curcumin (purity ≥ 98%, HPLC; Sigma-Aldrich, St. Louis, MO, USA) was dissolved in DMSO (final DMSO concentration < 0.1% in all groups) and administered 2 h prior to IL-1β stimulation. After 24 h of treatment, cells and culture supernatants were collected for downstream analyses. The curcumin concentrations (5 and 10 μM) were selected based on preliminary MTT viability assays showing >90% cell viability at both doses, and prior literature demonstrating that 5–10 μM curcumin achieves anti-inflammatory effects without significant cytotoxicity in chondrocyte models [12,22]. All experiments included six biological replicates per group (*n* = 6).

### 2.2. Untargeted Metabolomics Analysis

#### 2.2.1. Sample Preparation

Cells were washed with cold PBS, snap-frozen in liquid nitrogen, and extracted with pre-cooled methanol–water (4:1, *v*/*v*) at 150 μL per 10 mg pellet. Homogenization was performed at 70 Hz (3 × 20 s cycles), followed by incubation at −20 °C for 1 h. After centrifugation (16,000× *g*, 15 min, 4 °C), 300 μL of supernatant was collected. All samples were dried under nitrogen, reconstituted in 150 μL acetonitrile–water (1:1, *v*/*v*), vortexed, sonicated, and centrifuged; 120 μL of supernatant was used for UHPLC analysis. All experimental groups included six biological replicates (*n* = 6 per group, total 24 samples). A pooled quality control (QC) sample was prepared by mixing equal aliquots (10 μL) of all 24 experimental samples and was injected six times at regular intervals (every 10 samples) throughout the analytical batch to monitor system stability.

#### 2.2.2. UHPLC-QTOF-MS Analysis

Analysis was performed using a SCIEX Exion LC™ system coupled to a Triple TOF™ 5600+ mass spectrometer (SCIEX, Framingham, MA, USA) equipped with an Atlantis™ Premier BEH C18 AX column (100 × 2.1 mm, 1.7 μm; Waters, Milford, MA, USA) maintained at 40 °C. Mobile phases consisted of 0.1% formic acid in water (A) and acetonitrile (B) at a flow rate of 0.4 mL/min. The gradient program was: 0–1 min, 1% B; 1–10 min, 1–99% B; 10–13 min, 99% B; 13–14 min, 99–1% B; 14–17 min, 1% B. Injection volume was 10 μL. Mass spectrometric detection was performed in both electrospray ionization (ESI) positive and negative modes. Ion source parameters were: nebulizer gas (GS1) 55 psi, heater gas (GS2) 55 psi, curtain gas (CUR) 35 psi, source temperature 550 °C; ion spray voltage floating (ISVF) +5500 V (positive mode) and −4500 V (negative mode). MS/MS spectra were acquired via information-dependent acquisition (IDA) over *m*/*z* 100–1250.

#### 2.2.3. Data Processing and Metabolite Identification

Raw data files (.wiff) were converted to mzML format using ProteoWizard (v3.0). Peak alignment, retention time correction, feature extraction, and normalization were performed with XCMS Online (https://xcmsonline.scripps.edu, accessed on 21 January 2026). Metabolites were identified by accurate mass (<10 ppm) and MS/MS spectral matching against the Human Metabolome Database (HMDB, http://www.hmdb.ca, accessed on 21 January 2026) and METLIN (https://metlin.scripps.edu, accessed on 21 January 2026). Features present in ≥80% of samples within at least one group were retained for analysis. Data were normalized by median and Pareto-scaled prior to multivariate analysis.

#### 2.2.4. Quality Control Assessment

To evaluate analytical reproducibility, the relative standard deviation (RSD) of each metabolite was calculated across the six QC injections. Of the 214 detected metabolites, 199 (93.0%) exhibited RSD < 10%, 208 (97.2%) exhibited RSD < 15%, and 213 (99.5%) exhibited RSD < 20%, with a median RSD of 3.66% and mean RSD of 4.60% (Supplementary Figure S1). Principal component analysis (PCA) including all experimental samples and QC samples demonstrated that the QC samples clustered tightly in the PCA score plot, confirming minimal instrumental drift and robust data quality (Supplementary Figure S2).

#### 2.2.5. Multivariate Statistical Analysis

Multivariate statistical analyses were performed using the mixOmics R package (v6.18). Principal component analysis (PCA) assessed overall sample distribution and potential outliers. Partial least squares-discriminant analysis (PLS-DA) was employed to maximize group separation and identify discriminating metabolites. PLS-DA models were validated by leave-one-out cross-validation (LOOCV) to calculate Q^2^ (predictive ability) and 200-iteration permutation testing to assess model robustness and rule out overfitting. Model performance parameters are reported as R^2^X (cumulative explained variance in the X matrix), R^2^Y (cumulative explained variance in the Y matrix, model fit), and Q^2^ (predictive ability). All PLS-DA models demonstrated Q^2^ > 0.7 and passed permutation testing (all *p* ≤ 0.015; Supplementary Table S1, Figures S3–S7).

Stratified dimensionality reduction classified metabolites into six functional categories (amino acids, lipids, nucleotides, TCA cycle/energy, carbohydrates, and vitamins/cofactors). PCA and PLS-DA were performed for each category to identify pathway-specific effects of IL-1β stimulation and curcumin treatment. Significantly altered metabolites were defined by variable importance in projection (VIP) > 1 and Student’s *t*-test *p* < 0.05. Functional enrichment analysis was performed with MetaboAnalyst 5.0 (https://www.metaboanalyst.ca, 25 January 2026) using KEGG pathway mapping. Pathway topology analysis accounted for metabolite centrality within metabolic networks.

### 2.3. Transcriptomic Data Mining and Integration

Four publicly available gene expression datasets related to osteoarthritis were obtained from the Gene Expression Omnibus (GEO) database: GSE12021, GSE55235, GSE55457, and GSE82107 [23]. These datasets encompassed cartilage samples from OA patients and healthy controls, with a total of 168 OA samples and 65 normal control samples across all datasets. Raw expression data were downloaded and processed using Bioconductor packages (v3.21).

For Affymetrix microarray datasets (GSE12021, GSE55235, GSE55457), raw CEL files were background-corrected, normalized, and summarized using the robust multi-array average (RMA) algorithm implemented in the affy package (v1.72.0) [24]. For RNA-seq data (GSE82107), raw count data were normalized using the trimmed mean of M-values (TMM) method in the edgeR package (v3.40.2), followed by log_2_ transformation. Batch effects between datasets were corrected using the ComBat function in the sva package (v3.44.0) [25].

Differential gene expression analysis was performed using the limma package (v3.54.2) for microarray data and edgeR for RNA-seq data [26]. Genes with |log_2_ fold change| ≥ 1.5 and adjusted *p*-value < 0.05 (false discovery rate corrected) were considered significantly differentially expressed. A meta-analysis approach was employed to identify consensus differentially expressed genes (DEGs) across datasets using the RankProd (v3.4) method, which combines fold changes and *p*-values across studies while accounting for study-specific effects [27].

Metabolism-associated genes were identified by querying the Molecular Signatures Database (MSigDB, v7.4, https://www.gsea-msigdb.org, 25 January 2026) for genes annotated with metabolism-related Gene Ontology (GO) biological process terms and KEGG metabolism pathways. Genes appearing in two or more metabolism-related gene sets were classified as metabolism-associated. The intersection of these metabolism-associated genes with the OA consensus DEGs was then determined, identifying genes at the interface of metabolic pathways and OA-associated transcriptional dysregulation.

Functional enrichment analysis of DEGs was performed using the clusterProfiler R package (v4.15.0) [28]. Gene Ontology enrichment analysis was conducted for three categories: biological processes (BP), cellular components (CC), and molecular functions (MF). KEGG pathway enrichment analysis was performed using the enrichKEGG function with the human genome as the background. Benjamini–Hochberg false discovery rate correction was applied, and pathways with adjusted *p*-value < 0.05 were considered significantly enriched.

### 2.4. Machine Learning-Based Gene Signature Identification

Three complementary machine learning algorithms were employed to identify core genes from the 39-gene candidate set: LASSO regression, Random Forest, and XGBoost [29]. All analyses were performed in R (v4.2.3)using appropriate packages (glmnet (v4.1-7), randomForest (v4.7-1.1), and xgboost (v1.7.5.1), respectively).

For LASSO regression, 10-fold cross-validation was used to select the optimal regularization parameter λ. The lambda.1se value (the largest λ within one standard error of the minimum cross-validation error) was selected, following the standard “1-SE rule” that favors the most regularized model with performance statistically indistinguishable from the minimum-error model. This approach balances model complexity and predictive performance, reducing the risk of overfitting [30]. Genes retaining non-zero coefficients in the lambda.1se model were selected as LASSO-identified features.

Random Forest analysis was performed with 1000 trees (ntree = 1000) and the number of variables randomly sampled at each split (mtry) was set to the square root of the total number of predictor variables, following standard recommendations for classification tasks [31]. Feature importance was assessed using the Mean Decrease in the Gini impurity index. Genes with MeanDecreaseGini > 2 were retained [32], corresponding to the natural break point in the ranked importance distribution and ensuring retention of the most discriminative features.

XGBoost was implemented with 100 boosting rounds and 10-fold cross-validation. Feature importance was calculated using the gain metric, which quantifies the relative contribution of each feature to the reduction in loss function across all tree splits. Genes with a gain > 1 were selected, corresponding to the inflection point in the gain value distribution and retaining features with above-average contribution to model performance.

The intersection of genes selected by all three algorithms was determined using Venn diagram analysis, yielding a high-confidence six-gene signature. This consensus approach minimizes algorithm-specific biases and enhances the robustness of feature selection results.

### 2.5. Gene-Pathway Network Construction and Validation

#### 2.5.1. Network Analysis

A gene-pathway interaction network was constructed to visualize relationships between the six core genes and significantly enriched KEGG pathways. Network construction was performed using Cytoscape 3.9.0 [33], with genes and pathways represented as nodes and their associations as edges. Network topology was analyzed to identify central hub genes based on degree centrality and betweenness centrality measures.

To evaluate the discriminative performance of the six-gene signature, logistic regression was used to construct a classifier based on the expression levels of the six core genes. The combined GEO dataset (73 samples: 40 OA and 33 normal) was used for this analysis. The area under the receiver operating characteristic curve (AUC) was calculated using the pROC R package (v1.18) [34], with 95% confidence intervals determined by 2000 stratified bootstrap replicates. Sensitivity, specificity, and classification accuracy were determined at the optimal threshold identified by the Youden index maximization method.

#### 2.5.2. Quantitative PCR Validation

To validate the machine learning-identified gene signature and pathway analysis results, quantitative reverse transcription PCR (qRT-PCR) was performed on independent chondrocyte samples treated under identical conditions. Total RNA was extracted using TRIzol reagent (Invitrogen, Waltham, MA, USA) according to the manufacturer’s instructions. Complementary DNA synthesis was performed using 1 μg of total RNA with the PrimeScript RT reagent kit (Takara Bio, Kusatsu, Shiga, Japan). qRT-PCR was conducted using SYBR Green Master Mix (Applied Biosystems, Waltham, MA, USA) on a 7500 Real-Time PCR System. Primer sequences were designed using Primer-BLAST (National Center for Biotechnology Information, Bethesda, MD, USA) and validated for specificity (primer sequences in Table S1). Reaction conditions were: 95 °C for 10 min, followed by 40 cycles of 95 °C for 15 s and 60 °C for 1 min. Relative gene expression was calculated using the 2 ^−ΔΔCt^ method with GAPDH as the internal reference gene. Statistical analysis was performed using one-way ANOVA followed by Tukey’s post hoc test, with *p* < 0.05 considered statistically significant. All qRT-PCR experiments included six biological replicates and were performed in technical triplicate.

## 3. Results

### 3.1. Curcumin Ameliorates IL-1β-Induced Metabolic Disruptions in Chondrocytes

To evaluate the effects of curcumin against IL-1β-induced metabolic disruptions in human articular chondrocytes, untargeted metabolomics profiling was performed using UHPLC-QTOF-MS. A total of 214 metabolites were identified and quantified across all four experimental groups (Blank, Model, CurL, CurH). Comprehensive quality control assessment confirmed excellent analytical reproducibility: the median RSD of metabolite features across QC injections was 3.66%, with 99.5% of features exhibiting RSD < 20% (Supplementary Figure S1), and QC samples clustered tightly in the PCA score plot (Supplementary Figure S2), confirming minimal instrumental drift and robust data quality.

**Figure 1:**
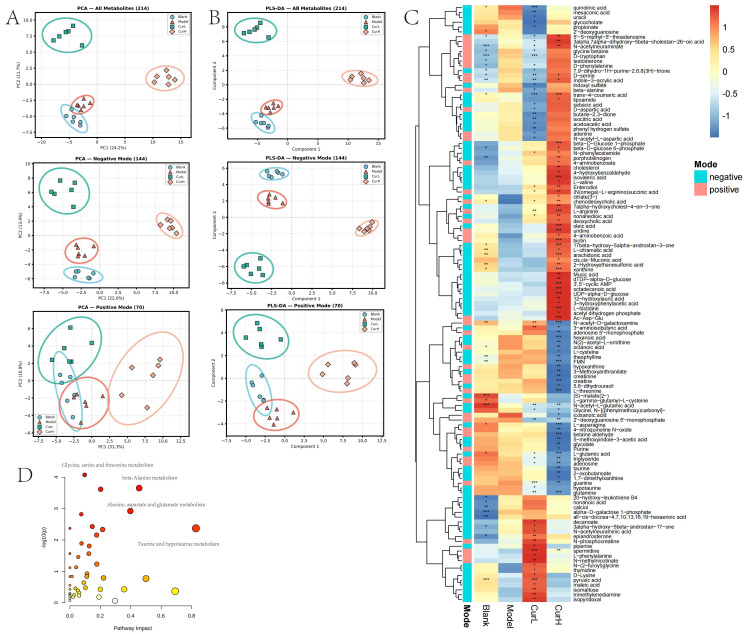
Metabolomic alterations in IL-1β-stimulated chondrocytes and curcumin-mediated rescue. (**A**) PCA score plots. (**B**) PLS-DA score plots. (**C**) Heatmap of differential metabolite abundance. (**D**) Pathway enrichment analysis of differential metabolites. Data represent mean ± SD; *n* = 6/group. * *p* < 0.05, ** *p* < 0.01, *** *p* < 0.001, vs. Model.

PCA score plots (Figure 1A) revealed distinct separation among experimental groups. PC1 accounted for 21.7% of the total variance, and PC2 accounted for 10.4%. The overall principal component distribution demonstrated that Model group samples were clearly separated from Blank controls along PC2, consistent with IL-1β-induced metabolic dysregulation. Notably, both the CurL and CurH groups were positioned intermediately between the Model and Blank groups, with CurH closer to Blank, suggesting a dose-dependent attenuation of IL-1β-induced metabolic perturbation.

Supervised PLS-DA (Figure 1B) further validated these inter-group differences. The PLS-DA models for all five pairwise comparisons demonstrated robust performance with Q^2^ values exceeding 0.7 and all passing 200-iteration permutation testing (*p* ≤ 0.005–0.015; Supplementary Table S1, Figures S3–S7). The clear separation observed in PLS-DA score plots confirmed that IL-1β treatment induced substantial metabolic alterations, and curcumin treatment—particularly at the high dose (10 μM)—was associated with metabolic profiles shifted toward the Blank condition.

To delineate pathway-specific contributions, stratified PCA and PLS-DA models were constructed across six functional metabolite categories (Supplementary Figure S8). Among amino acid metabolism (64 metabolites), the most pronounced inter-group discrimination was observed, followed by lipids (58 metabolites) and TCA cycle/energy metabolism (29 metabolites). Nucleotides (21 metabolites), carbohydrates (20 metabolites), and vitamins/cofactors (6 metabolites) showed comparatively limited inter-group discrimination.

The heatmap (Figure 1C) visualized expression patterns across 123 significantly altered metabolites (VIP > 1, *p* < 0.05). The Model group showed marked reductions in key TCA cycle intermediates—including propionate, pyruvic acid, (S)-malate^2−^, and citrate^3−^—suggesting IL-1β-induced impairment of mitochondrial energy metabolism. Amino acid-related metabolites, including glutamine, L-glutamic acid, and N-acetylneuraminic acid, were also significantly dysregulated, consistent with inflammatory perturbation of amino acid homeostasis. Curcumin treatment attenuated these aberrations in a dose-dependent fashion: CurH significantly elevated propionate, (S)-malate^2−^, and glutamine levels (*p* < 0.05), while CurL effectively normalized butane-2,3-dione, isovaleric acid, and 3-aminoisobutyric acid (*p* < 0.05).

Pathway enrichment analysis (Figure 1D) revealed that differentially abundant metabolites were predominantly enriched in beta-alanine metabolism, arginine biosynthesis, alanine/aspartate/glutamate metabolism, pantothenate and CoA biosynthesis, and purine metabolism. These pathways are functionally linked to mitochondrial energy metabolism, antioxidant defense (carnosine synthesis via beta-alanine), and nucleotide homeostasis.

### 3.2. Transcriptomic Analysis Identifies OA-Associated Metabolic Genes

**Figure 2:**
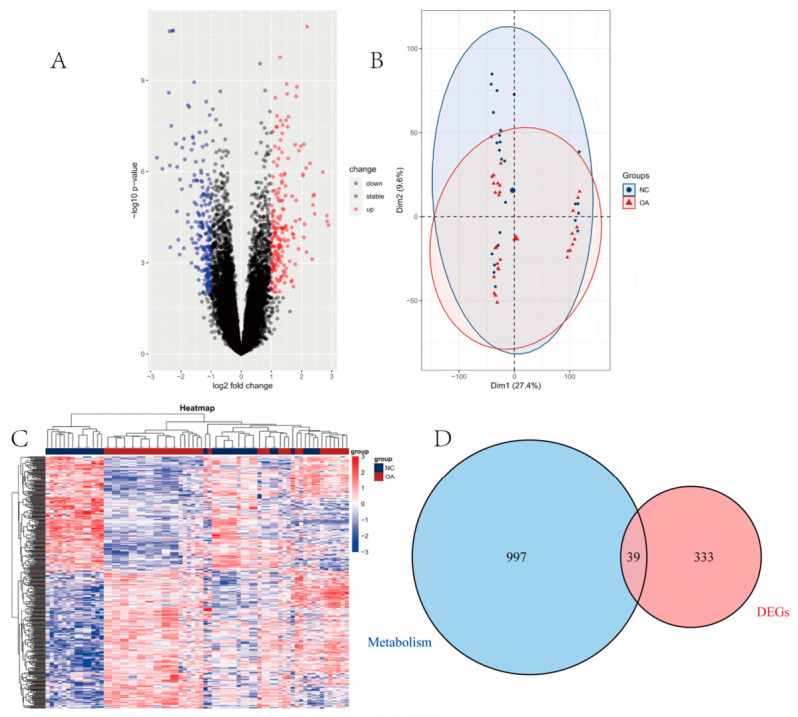
Transcriptomic landscape of osteoarthritis reveals metabolism-linked DEGs. (**A**) Volcano plot of integrated GEO datasets (GSE12021, GSE55235, GSE55457, GSE82107) showing DEGs in OA vs. normal control (NC). (**B**) PCA plot demonstrating separation between OA (red) and NC (blue) groups. (**C**) Heatmap of gene expression patterns in OA vs. NC; red = high expression, blue = low expression. (**D**) Venn diagram identifying 39 overlapping genes between metabolism-associated predicted genes and DEGs.

To delineate the gene expression landscape in OA, we performed integrative transcriptomic analysis of four GEO datasets (GSE12021, GSE55235, GSE55457, GSE82107) comprising a total of 40 OA and 37 normal human articular cartilage samples. After batch effect correction and robust rank aggregation, 486 consensus DEGs were identified between OA and normal cartilage (|log_2_ FC| ≥ 1.5, adjusted *p* < 0.05). The volcano plot (Figure 2A) visualized the distribution of DEGs, while the PCA plot (Figure 2B) showed clear separation between OA and normal samples along the first discriminant dimension (Dim1, 27.4% of total variance). The heatmap (Figure 2C) demonstrated consistent gene expression patterns distinguishing the two groups.

Metabolism-associated genes were identified by querying the Molecular Signatures Database (MSigDB) for metabolic gene sets (see Section 2.3). The intersection of these metabolism-associated genes with the 486 OA consensus DEGs yielded 39 genes (Figure 2D), indicating that a substantial subset of OA-dysregulated genes is directly linked to metabolic pathways. These 39 genes represent candidate mediators at the interface of metabolic dysfunction and OA pathogenesis.

### 3.3. Functional Enrichment Analysis Implicates Pathways of Curcumin Anti-OA

**Figure 3:**
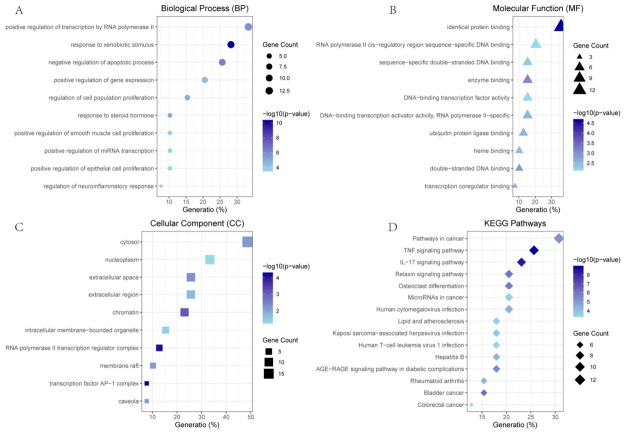
GO and KEGG enrichment of 39 overlapping metabolism-DEGs. (**A**) Top 10 enriched biological processes (BP). (**B**) Top 10 molecular functions (MF). (**C**) Top 10 cellular components (CC). (**D**) Top 15 KEGG pathways.

To elucidate the potential mechanisms by which curcumin may exert therapeutic effects in OA, we conducted GO and KEGG pathway enrichment analyses on the 39 overlapping genes identified between metabolism-associated predicted genes and DEGs. The analysis revealed significant enrichment in key biological processes (Figure 3A) including positive regulation of transcription by RNA polymerase II, response to xenobiotic stimulus, negative regulation of apoptotic process, positive regulation of gene expression, and response to steroid hormone. Enriched molecular functions (Figure 3B) included identical protein binding, enzyme binding, double-stranded DNA binding, transcription coregulator binding, and heme binding. Cellular components (Figure 3C) were notably associated with the transcription factor AP-1 complex, RNA polymerase II transcription regulator complex, chromatin, cytosol, and extracellular region. KEGG pathway (Figure 3D) analysis further demonstrated significant involvement in Pathways in cancer, TNF signaling pathway, IL-17 signaling pathway, Relaxin signaling pathway, osteoclast differentiation, AGE-RAGE signaling pathway in diabetic complications, and Rheumatoid arthritis. These enriched terms and pathways are closely related to inflammation, metabolic regulation, and extracellular matrix remodeling—processes known to be central to OA progression and previously reported as targets of curcumin. Therefore, these results suggest that the therapeutic benefits of curcumin in OA may be mediated through modulation of these functional pathways and biological processes, particularly by influencing inflammatory signaling, cellular stress responses, and transcriptional regulation.

### 3.4. Machine Learning Algorithms Identify a Core Inflammatory-Metabolic Gene Signature

**Figure 4:**
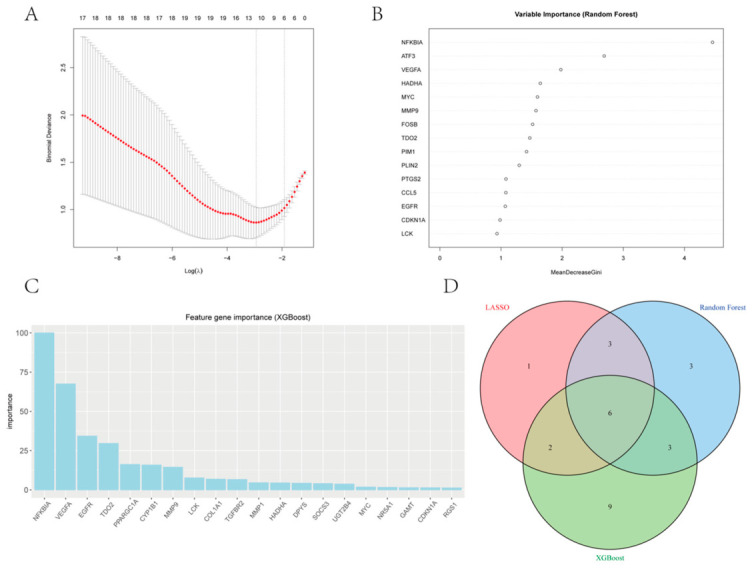
Machine learning-driven identification of core genes. (**A**) LASSO regression coefficient profiles retaining 12 genes with non-zero coefficients. (**B**) Random Forest gene selection identifying 15 top genes. (**C**) XGBoost feature importance ranking of 10 genes. (**D**) Venn diagram of machine learning results identifying core genes.

To identify the most informative core genes within the 39-gene candidate set, we applied three complementary machine learning algorithms. LASSO regression with 10-fold cross-validation (Figure 4A) retained 12 genes with non-zero coefficients at lambda.1se, balancing model parsimony with predictive performance. Random Forest analysis (Figure 4B) retained 18 genes with MeanDecreaseGini > 2, representing features above the natural break point in the importance distribution. XGBoost (Figure 4C) selected 15 genes with importance gain > 1, retaining features with above-average contribution to model performance.

The intersection of genes selected by all three algorithms, visualized through Venn diagram analysis (Figure 4D), revealed a robust consensus six-gene core signature: *NFKBIA*, *MMP9*, *LCK*, *TDO2*, *HADHA*, and *VEGFA*. *NFKBIA* encodes IκBα, the primary endogenous inhibitor of NF-κB signaling. *MMP9* encodes matrix metalloproteinase-9, a key enzyme in cartilage extracellular matrix degradation. *LCK* encodes lymphocyte-specific protein tyrosine kinase, implicated in inflammatory signaling. *TDO2* encodes tryptophan 2,3-dioxygenase, a rate-limiting enzyme in tryptophan catabolism. *HADHA* encodes the α-subunit of mitochondrial trifunctional protein, essential for fatty acid β-oxidation. *VEGFA* encodes vascular endothelial growth factor A, involved in angiogenesis and inflammation.

To evaluate the discriminative performance of this six-gene signature, logistic regression modeling was performed on the combined GEO dataset. Each of the six individual genes showed favorable diagnostic performance to discriminate OA cartilage from normal cartilage samples, with all respective AUC values exceeding 0.75 (Supplementary Figure S9). In an independent validation subset (*n* = 73), the classifier achieved an AUC of 0.98, a sensitivity of 95.0%, and a specificity of 93.9% (Supplementary Figure S10), confirming that the six-gene signature captures biologically meaningful differences between OA and normal cartilage. The higher AUC in the validation subset likely reflects reduced inter-dataset heterogeneity.

### 3.5. Curcumin Modulates IL-17/TNF Signaling Pathways and Validates Core Gene Expression

**Figure 5:**
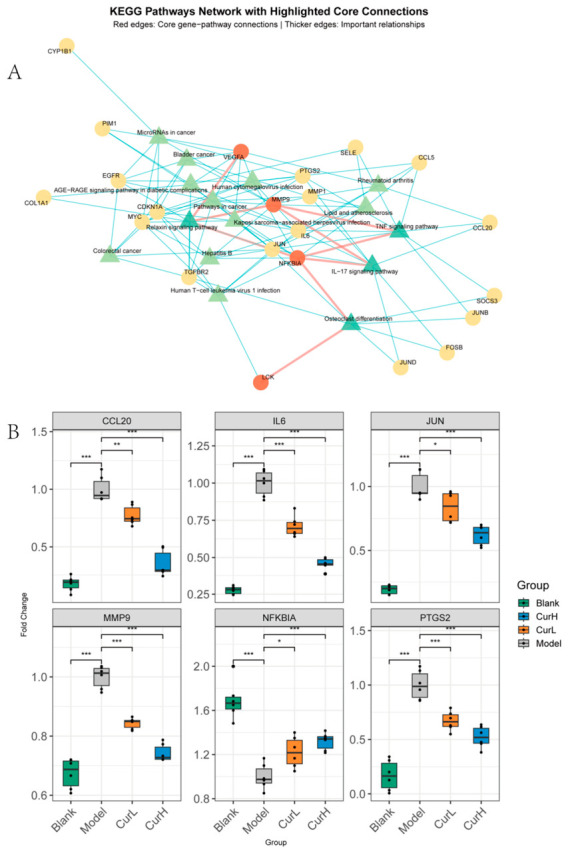
Curcumin influences IL-17/TNF signaling through *NFKBIA* upregulation. (**A**) Gene-Pathway Interaction Network. Network diagram showing the interactions between the core genes and significantly enriched KEGG pathways (IL-17 signaling pathway, TNF signaling pathway). Nodes represent genes or pathways, and edges represent associations. Key hub genes like *NFKBIA*, *VEGFA*, and *MMP9* are central to the network. (**B**) qRT-PCR validation in IL-1β-induced chondrocytes: Curcumin dose-dependently reversed IL-1β-induced overexpression of *JUN*, *IL6*, *PTGS2*, and *CCL20* and restored *NFKBIA* levels. Data represent mean ± SD; *n* = 6/group. * *p* < 0.05, ** *p* < 0.01, *** *p* < 0.001, vs. Model.

To elucidate the regulatory context of the six core genes, we constructed a gene-pathway interaction network integrating these genes with significantly enriched KEGG pathways (Figure 5A). Network topological analysis identified *NFKBIA* as a highly connected node based on degree centrality and betweenness centrality measures. The network topology indicated that *NFKBIA*, *VEGFA*, *MMP9*, *PTGS2*, *CCL5*, *JUN*, and *IL6* are linked to IL-17 and TNF inflammatory signaling cascades—both pathways central to OA synovitis and cartilage degradation.

Using qRT-PCR, we experimentally validated curcumin’s effects on this signaling axis in IL-1β-challenged human articular chondrocytes (Figure 5B). In the IL-1β-induced OA model, mRNA levels of inflammatory mediators were significantly elevated relative to Blank controls: *JUN* (log2FC = 2.38, *p* < 0.001), *IL6* (log2FC = 1.85, *p* < 0.001), *PTGS2* (log2FC = 2.56, *p* < 0.001), *CCL20* (log2FC = 2.51, *p* < 0.001), and *MMP9* (log2FC = 0.57, *p* < 0.001). Conversely, *NFKBIA* mRNA was significantly reduced (log2FC = −0.76, *p* < 0.001), consistent with NF-κB pathway activation.

High-dose curcumin treatment (CurH, 10 μM) was associated with significant attenuation of these IL-1β-induced changes. Compared to the Model group, CurH treatment significantly downregulated *JUN* (log2FC = −0.69, *p* < 0.001), *IL6* (log2FC = −1.13, *p* < 0.001), *PTGS2* (log2FC = −0.94, *p* < 0.001), *CCL20* (log2FC = −1.51, *p* < 0.001), and *MMP9* (log2FC = −0.43, *p* < 0.001). Moreover, *NFKBIA* expression was significantly upregulated in the CurH group relative to Model (log2FC = 0.40, *p* < 0.001).

## 4. Discussion

This study provides a systems-level characterization of curcumin’s effects on IL-1β-challenged human articular chondrocytes, revealing coordinated alterations across metabolic and transcriptomic networks. By integrating untargeted metabolomics, multi-cohort transcriptomic analysis, and consensus machine learning, our findings suggest that curcumin’s pleiotropic effects may involve concurrent modulation of mitochondrial bioenergetics, amino acid metabolism, and inflammatory gene networks—providing a more comprehensive framework for understanding the compound’s mechanisms beyond the limitations of single-target analyses. However, as discussed below, these findings must be interpreted within the context of curcumin’s significant pharmacological limitations.

Metabolic effects. Our metabolomic analyses demonstrate that IL-1β stimulation induces pronounced metabolic disruptions in chondrocytes, most notably the depletion of TCA cycle intermediates (propionate, malate, citrate, pyruvic acid) and alterations in amino acid profiles. These findings are consistent with the established paradigm of inflammatory metabolic reprogramming, wherein pro-inflammatory cytokines promote a glycolytic shift at the expense of mitochondrial oxidative metabolism [7,35]. The observed reductions in TCA cycle intermediates may reflect diminished mitochondrial flux through the electron transport chain, potentially contributing to the bioenergetic crisis that characterizes OA chondrocytes [36].

Curcumin treatment—particularly at the 10 μM dose—was associated with substantial restoration of these metabolic alterations. The clear separation of CurH group samples from Model group samples in both PCA and PLS-DA spaces, along with the dose-dependent positioning of CurL and CurH groups between Model and Blank conditions, supports an association between curcumin treatment and attenuation of IL-1β-induced metabolic dysregulation. The restoration of TCA cycle intermediates and amino acid levels (including glutamine and glutamate) is consistent with effects on mitochondrial bioenergetics and anaplerotic pathways [37].

The enrichment of the beta-alanine metabolism pathway may relate to antioxidant defense mechanisms through carnosine synthesis, as beta-alanine is a rate-limiting precursor for carnosine—a dipeptide with documented free radical-scavenging properties [38]. Curcumin’s association with elevated beta-alanine levels could reflect an indirect antioxidant mechanism complementing its direct radical-scavenging activity. Similarly, alterations in arginine biosynthesis pathway metabolites suggest modulation of nitrogen metabolism and potentially nitric oxide signaling, which plays dual roles in cartilage homeostasis and catabolism [39]. Curcumin treatment was also associated with changes in purine and pyrimidine metabolism, potentially reflecting effects on nucleotide homeostasis and cellular energy charge. The observed changes in fatty acid metabolism may indicate effects on membrane lipid composition and β-oxidation, consistent with the identification of HADHA in our machine learning signature.

Machine learning-guided gene signature. The convergence of three independent machine learning algorithms (LASSO, Random Forest, XGBoost) on a six-gene signature (*NFKBIA, MMP9, LCK, TDO2, HADHA, VEGFA*) provides computational evidence that these genes may represent an integrated inflammatory-metabolic network relevant to OA pathogenesis. Importantly, this multi-algorithm consensus approach reduces the risk of algorithm-specific bias and enhances confidence in the selected features [32]. The six genes span diverse but interconnected biological functions: NF-κB regulation (*NFKBIA*), extracellular matrix degradation (*MMP9*), immune signaling (*LCK*), amino acid catabolism (*TDO2*), fatty acid oxidation (*HADHA*), and angiogenesis/inflammation (*VEGFA*)—collectively capturing key nodes at the metabolic-inflammatory interface.

*NFKBIA*, which encodes IκBα (the primary endogenous inhibitor of NF-κB signaling), emerged as a highly connected node in the gene-pathway interaction network. Our qRT-PCR data confirmed that IL-1β stimulation reduced *NFKBIA* mRNA levels and that curcumin treatment was associated with restoration of *NFKBIA* expression in a dose-dependent manner. This is consistent with the canonical model wherein IκBα degradation permits NF-κB nuclear translocation, and de novo IκBα synthesis serves as a negative feedback mechanism to terminate NF-κB-driven transcription [40]. However, we emphasize that our current evidence is limited to mRNA-level measurements, and a definitive demonstration of NFKBIA as a regulatory mediator would require: (1) protein-level confirmation of IκBα expression changes; (2) direct assessment of NF-κB (p65) nuclear translocation and DNA-binding activity; and (3) functional validation through *NFKBIA* gene silencing or overexpression studies. Pending such evidence, we consider *NFKBIA* a promising candidate mediator of the observed transcriptional responses rather than a proven regulatory hub.

*MMP9*, identified by all three algorithms, encodes matrix metalloproteinase-9, a gelatinase critically involved in cartilage type II collagen and aggrecan degradation [41]. Our qRT-PCR validation confirmed that IL-1β significantly upregulated MMP9 expression (log2FC = 0.57, *p* < 0.001) and that high-dose curcumin substantially suppressed this induction (log2FC = −0.43 vs. Model, *p* < 0.001). To our knowledge, this represents the first experimental validation of MMP9 within the context of this six-gene signature. The transcriptional modulation of MMP9 by curcumin, consistent with the known role of NF-κB in MMP9 transcriptional regulation, supports the hypothesis that curcumin’s effects on matrix-degrading enzymes may be mediated, at least in part, through modulation of NF-κB-dependent transcription.

Among the remaining signature genes, *LCK* (lymphocyte-specific protein tyrosine kinase) has been implicated in T-cell receptor signaling and inflammatory arthritis [42]; *TDO2* (tryptophan 2,3-dioxygenase) catalyzes the rate-limiting step of tryptophan degradation along the kynurenine pathway, with downstream metabolites exhibiting immunomodulatory properties [43]; *HADHA* (hydroxyacyl-CoA dehydrogenase trifunctional multienzyme complex subunit α) is essential for mitochondrial long-chain fatty acid β-oxidation, linking metabolic function to inflammatory regulation [44]; and *VEGFA* (vascular endothelial growth factor A) contributes to synovial angiogenesis and inflammation in OA [45]. While these genes were not independently validated at the transcript level in the present study, their mechanistic connections to both metabolic and inflammatory pathways make them compelling candidates for future investigation.

Limitations and caveats. Several important limitations must be carefully considered when interpreting the findings of this study.

First, regarding curcumin as a chemical probe: Curcumin is classified as a Pan-Assay Interference Compound (PAINS) and an Invalid Metabolic Panacea (IMPS) candidate [13,14]. Its well-documented liabilities include poor aqueous solubility (approximately 11 ng/mL at neutral pH), rapid degradation under physiological conditions (half-life < 30 min at pH 7.4), low oral bioavailability (<1% of ingested dose reaching systemic circulation), and promiscuous interactions with multiple protein targets and assay detection systems [13,14]. These properties collectively raise the possibility that some of the observed effects—particularly in cell-free or single-endpoint assays—may reflect non-specific interactions rather than specific target engagement. In the present study, we have attempted to mitigate these concerns through the use of orthogonal, multi-platform approaches (UHPLC-QTOF-MS metabolomics, microarray transcriptomics, and targeted qRT-PCR). Nevertheless, the potential for assay interference cannot be entirely excluded, and independent replication using alternative methodologies and structurally distinct NF-κB pathway modulators is warranted. Furthermore, as noted by Aggarwal et al., turmeric contains hundreds of potentially bioactive compounds, and “curcumin-free” turmeric preparations retain anti-inflammatory activity [15]. Our study employed purified curcumin (>98%), and the extent to which the observed effects are attributable to curcumin specifically—versus potential minor turmeric constituents, degradation products, or non-specific mechanisms—merits consideration. We emphasize that this study characterizes mechanistic associations rather than demonstrating specific drug-target interactions, and the results should not be interpreted as evidence of clinical efficacy. The translational gap between these in vitro mechanistic findings and potential clinical applications remains substantial, constrained fundamentally by curcumin’s pharmacokinetic and physicochemical limitations.

Second, regarding the experimental model: Our in vitro chondrocyte model using acute IL-1β stimulation (24 h) does not fully recapitulate the complex tissue architecture, multifactorial etiology, and chronic time course of human OA. OA cartilage exists within a biomechanically active joint environment influenced by mechanical loading, synovial inflammation, subchondral bone remodeling, and systemic metabolic factors—none of which are captured in monolayer chondrocyte cultures. Moreover, the IL-1β model represents acute inflammatory stress, whereas clinical OA involves chronic, low-grade inflammation with contributions from multiple cytokines (IL-1β, TNF-α, IL-6), damage-associated molecular patterns (DAMPs), and mechano-inflammatory pathways. The transcriptomic component of our study analyzed human OA cartilage samples (from joint replacement surgery, representing end-stage disease), while the experimental validation employed an acute IL-1β-challenged chondrocyte model. These represent fundamentally different biological contexts: chronic degenerative tissue versus acute in vitro stimulation. Direct extrapolation between these systems should therefore be made with caution. Future studies should validate these findings in three-dimensional cartilage explant models, co-culture systems incorporating synovial fibroblasts, mechanically loaded chondrocyte systems, and ultimately in well-characterized clinical samples spanning different OA stages.

Third, regarding validation scope: The machine learning analysis identified a six-gene consensus signature, but comprehensive experimental validation at the transcript level was performed for *NFKBIA*, *MMP9*, and inflammatory markers (*JUN, IL6, PTGS2, CCL20*), while *LCK*, *TDO2*, *HADHA*, and *VEGFA* were not independently validated. Furthermore, all validation was conducted at the mRNA level; corresponding protein expression data, enzyme activity measurements, and functional perturbation studies (siRNA knockdown or cDNA overexpression) are needed to establish causal relationships between these genes and the observed metabolic and inflammatory phenotypes. The gene-pathway interaction network, while informative for hypothesis generation, reflects computational predictions that require experimental verification.

Fourth, the temporal dynamics of curcumin’s effects remain unexplored. Our study examined a single 24 h time point, but the kinetics of curcumin’s cellular uptake, its intracellular stability, and the time course of transcriptional and metabolic responses—including potential adaptive or compensatory mechanisms—require investigation through time-course experiments.

## 5. Conclusions

This study suggests that curcumin may influence metabolic dysfunction, inflammatory gene expression, and matrix-degrading enzyme production in OA through coordinated effects on multiple cellular networks. The integrated multi-omics analysis revealed that curcumin treatment was associated with restoration of TCA cycle intermediates, normalization of amino acid metabolism, and modulation of a six-gene inflammatory-metabolic signature. Among these genes, *NFKBIA* and MMP9 received experimental validation at the transcript level, positioning *NFKBIA* as a promising candidate mediator of the observed gene regulatory responses. However, these mechanistic interpretations must be considered in the context of curcumin’s well-documented pharmacological limitations—including its PAINS classification, poor bioavailability, and chemical instability—which substantially constrain direct clinical translation. These findings, therefore, provide a mechanistic framework that may inform the development of more drug-like molecules targeting the metabolic-inflammatory interface in OA, rather than advocating for curcumin itself as a clinical candidate. Future studies incorporating protein-level validation, functional gene perturbation experiments, and pharmacokinetic optimization will be essential to translate these mechanistic insights into therapeutic applications.

## Data Availability

The data presented in this study are available on request from the corresponding author due to ethical restrictions.

## Supplementary Materials

The following supporting information can be downloaded at: https://www.mdpi.com/article/10.3390/metabo16070506/s1, Table S1: PLS-DA model validation parameters for all pairwise comparisons. Table S2: Primer sequences used for qRT-PCR validation. Figure S1: Quality control: Relative standard deviation (RSD) distribution of 214 detected metabolites across six QC injections. Figure S2: PCA score plot including all experimental samples and QC samples. Figure S3. PLS-DA score plots (left panels) and corresponding 200-iteration permutation test results (right panels) for Model vs Blank. Figure S4. PLS-DA score plots (left panels) and corresponding 200-iteration permutation test results (right panels) for Cur-L vs Model. Figure S5. PLS-DA score plots (left panels) and corresponding 200-iteration permutation test results (right panels) for Cur-H vs Model. Figure S6. PLS-DA score plots (left panels) and corresponding 200-iteration permutation test results (right panels) for Cur-L vs Blank. Figure S7. PLS-DA score plots (left panels) and corresponding 200-iteration permutation test results (right panels) for Cur-H vs Blank. Figure S8: Separate ROC curves for individual constituent genes. Figure S9: ROC curve for the combined six-gene diagnostic signature. Figure S10: PCA & PLS-DA-Stratified by Metabolite Category.

## Abbreviations

The following abbreviations are used in this manuscript:

OA	osteoarthritis
IL-1β	interleukin-1 beta
TNF-α	tumor necrosis factor-alpha
NSAIDs	non-steroidal anti-inflammatory drugs
NF-κB	nuclear factor kappa-light-chain-enhancer of activated B cells
LC-MS	liquid chromatography-mass spectrometry
PBS	phosphate-buffered saline
UHPLC	ultra-high-performance liquid chromatography
QTOF-MS	quadrupole time-of-flight mass spectrometry
ESI	electrospray ionization
PQN	probabilistic quotient normalization
PLS-DA	partial least squares-discriminant analysis
VIP	variable importance in projection
HMDB	Human Metabolome Database
KEGG	Kyoto Encyclopedia of Genes and Genomes
GEO	Gene Expression Omnibus
RMA	robust multi-array average
TMM	trimmed mean of M-values
DEGs	differentially expressed genes
LASSO	least absolute shrinkage and selection operator
XGBoost	eXtreme Gradient Boosting
qRT-PCR	quantitative reverse transcription polymerase chain reaction
TCA	tricarboxylic acid
GO	Gene Ontology
PCA	principal component analysis
NC	normal control
BP	biological processes
MF	molecular functions
CC	cellular components
AP-1	activator protein-1
AGE-RAGE	advanced glycation end-products—receptor for advanced glycation end-products
NFKBIA	nuclear factor of kappa light polypeptide gene enhancer in B-cells inhibitor, alpha
MMP9	matrix metallopeptidase 9
LCK	lymphocyte-specific protein tyrosine kinase
TDO2	tryptophan 2,3-dioxygenase
HADHA	hydroxyacyl-CoA dehydrogenase trifunctional multienzyme complex subunit alpha
VEGFA	vascular endothelial growth factor A
