# Supplementary material for: Restoring Metabolic-Inflammatory Homeostasis: Curcumin’s Multi-Layered Defense Against Chondrocyte Dysfunction
Source: Metabolites. 2026 Jul 19;16(7):506. doi: 10.3390/metabo16070506 (PMC13414324; doi:10.3390/metabo16070506)
Supplement: Supplementary file 1 [file metabolites-16-00506-s001.zip › metabolites-4360199-supplementary.pdf]

Table S1. PLS-DA model validation parameters for all pairwise comparisons.

Comparison	R^2X	R^2Y	Q^2	p (200 permutations)
Model vs Blank	0.3037	0.9975	0.7321	0.0050
Cur-L vs Model	0.3341	0.9981	0.8366	0.0149
Cur-H vs Model	0.3830	0.9986	0.9049	0.0050
Cur-L vs Blank	0.3831	0.9984	0.9128	0.0050
Cur-H vs Blank	0.4276	0.9979	0.9487	0.0050

Table S2. Primer sequences used for qRT-PCR validation.

Gene	Forward	Reverse
NFKBIA	CTCCGAGACTTTCGAGGAAATAC	GCCATTGTAGTTGGTAGCCTTCA
VEGFA	AGGGCAGAATCATCACGAAGT	AGGGTCTCGATTGGATGGCA
MMP9	TGTACCGCTATGGTTACACTCG	GGCAGGGACAGTTGCTTCT
PTGS2	CTGGCGCTCAGCCATACAG	CGCACTTATACTGGTCAAATCCC
CCL5	CCAGCAGTCGTCTTTGTCAC	CTCTGGGTTGGCACACACTT
JUN	TGTACCGACTGAGAGTTCTTGA	ACAGAGCGAGTGAAAATGTGTAT
IL6	GAAGAGCGCCGCTGAGAAT	GTGCAGAGGGTTTAATGTCAACT

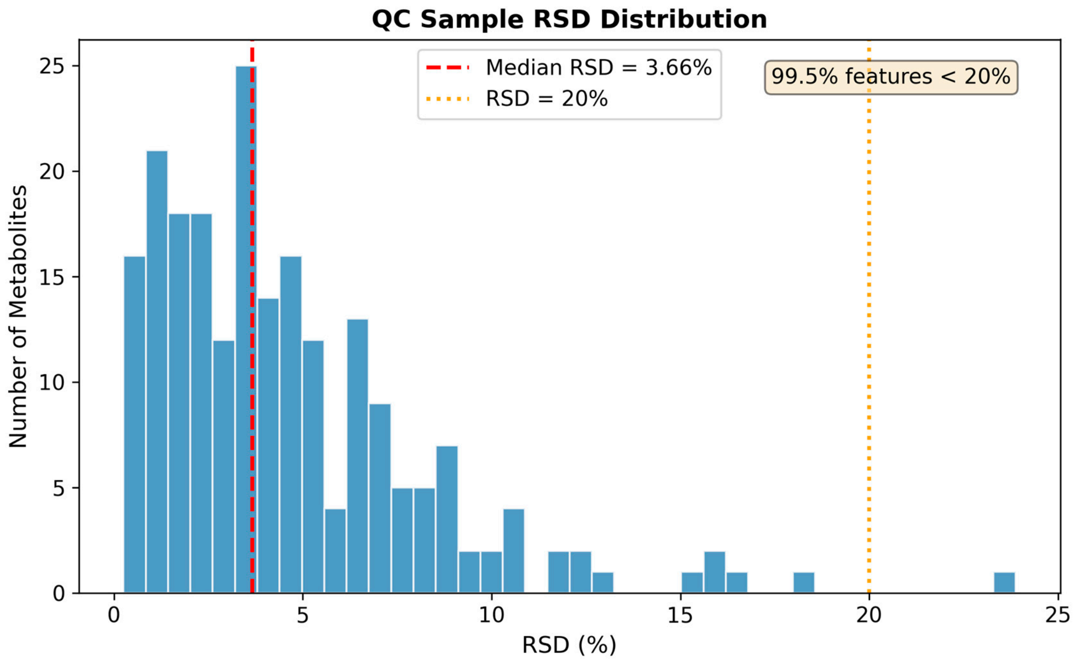

Figure S1. Quality control: Relative standard deviation (RSD) distribution of 214 detected metabolites across six QC injections.

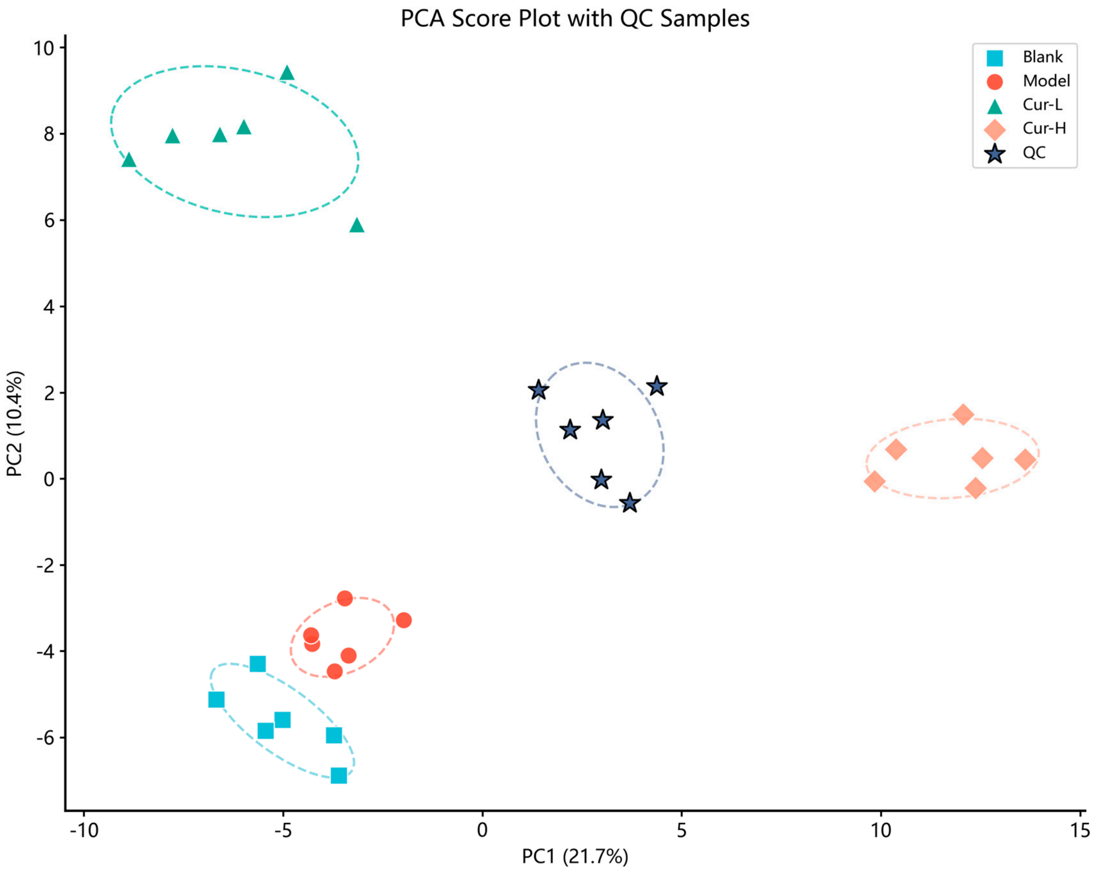

Figure S2. PCA score plot including all experimental samples and QC samples.

Figures S3–S7. PLS-DA score plots (left panels) and corresponding 200-iteration permutation test results (right panels)

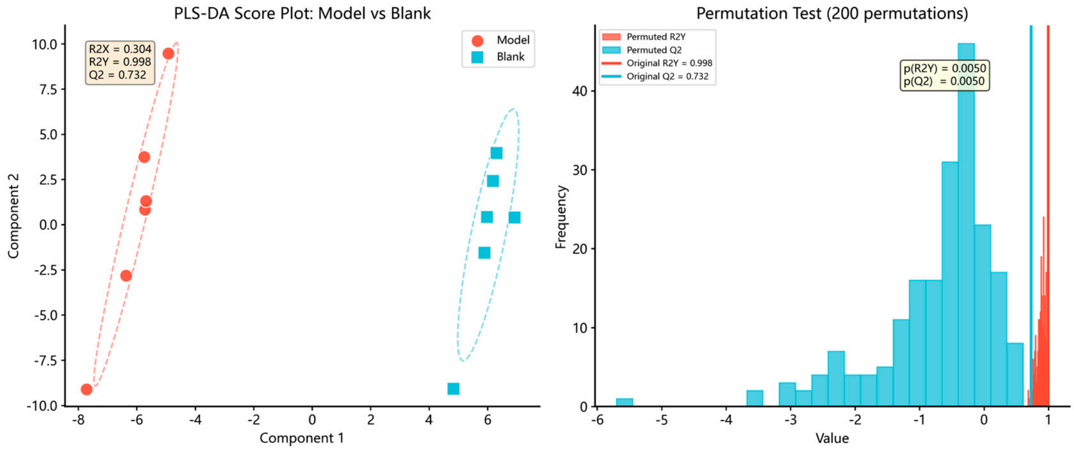

(S3) Model vs Blank;

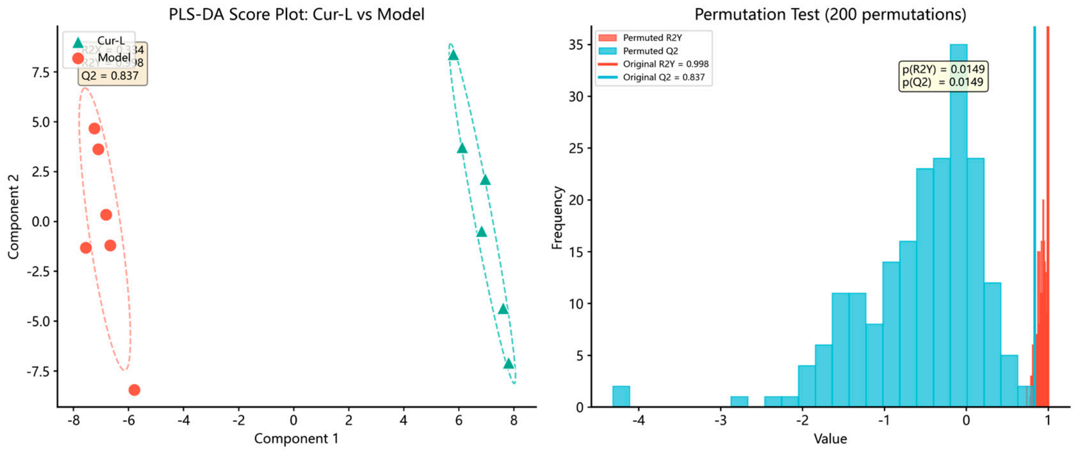

(S4) Cur-L vs Model;

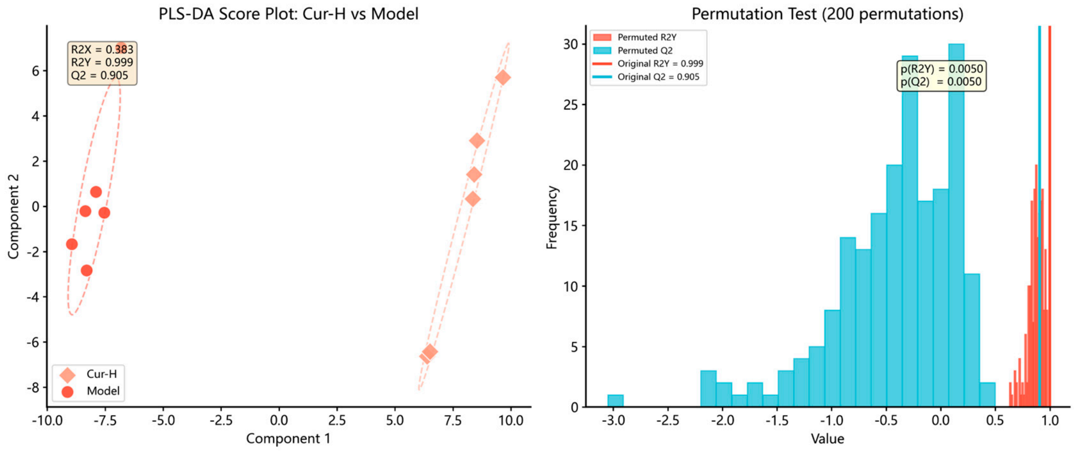

(S5) Cur-H vs Model;

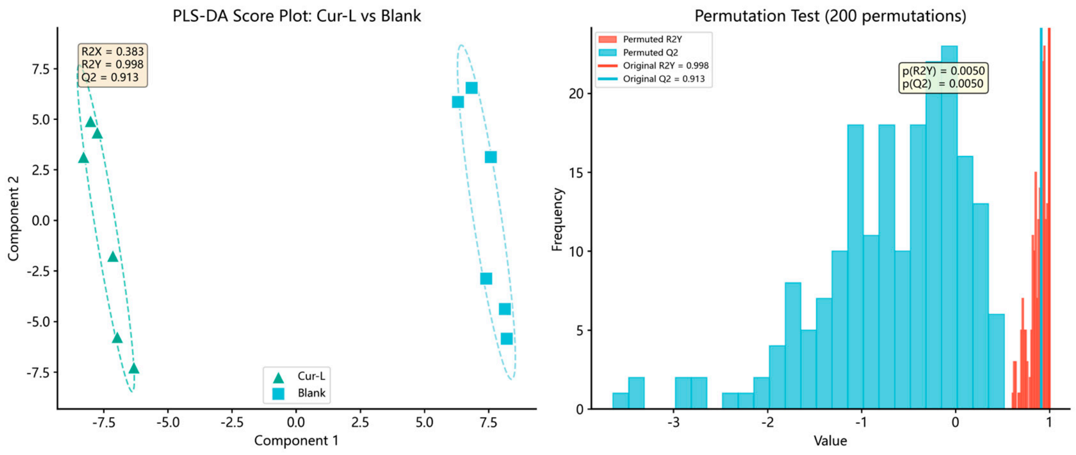

(S6) Cur-L vs Blank;

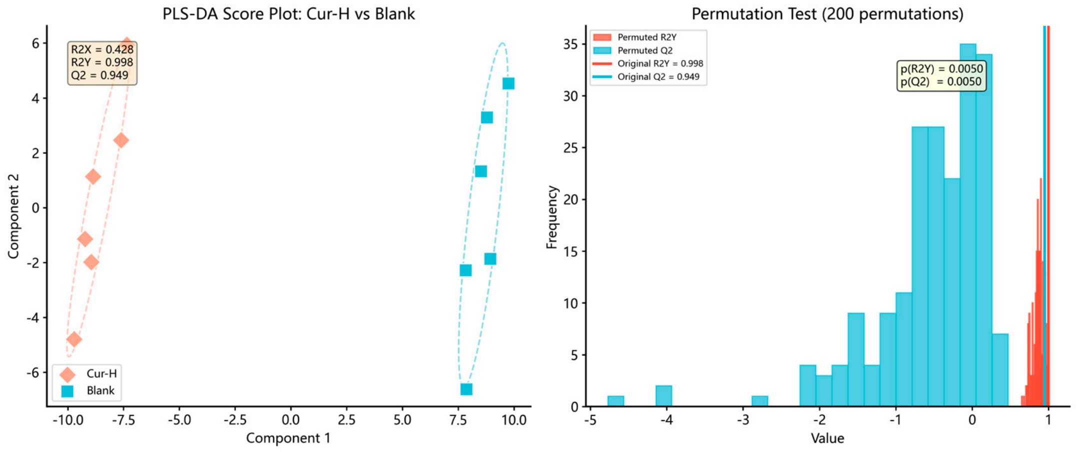

(S7) Cur-H vs Blank.

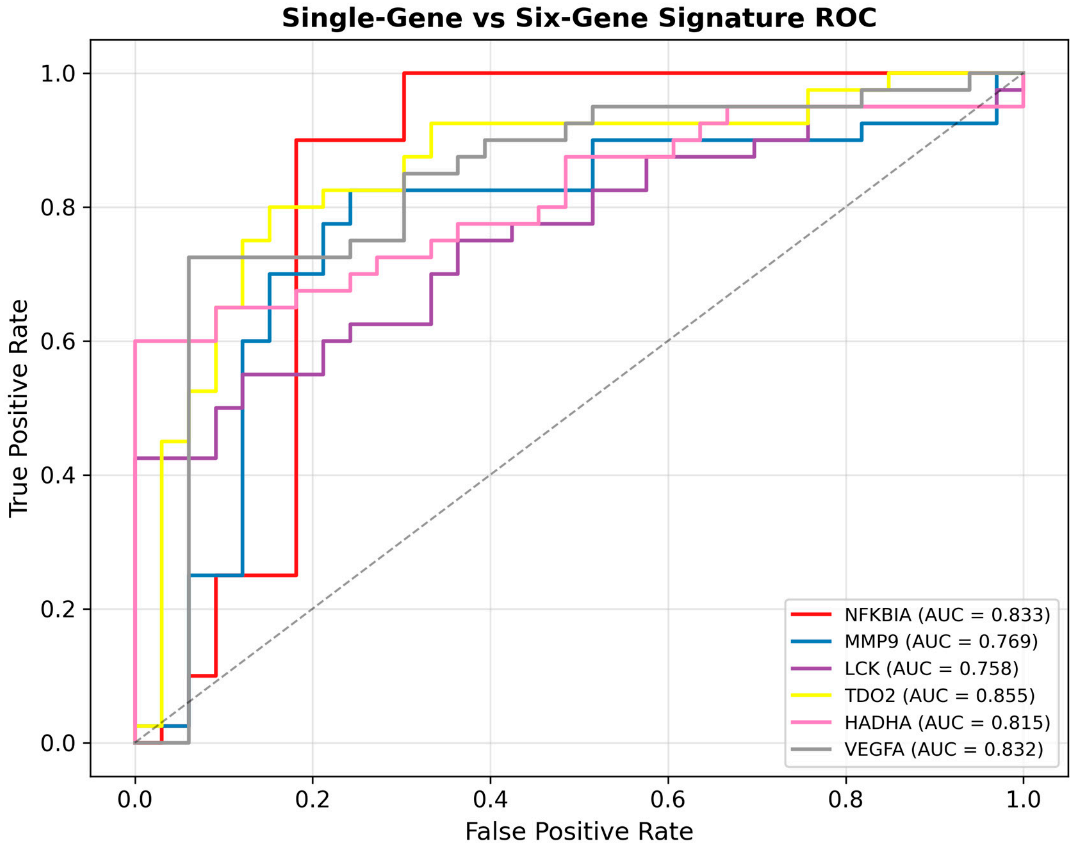

Figure S8. Separate ROC curves for individual constituent genes.

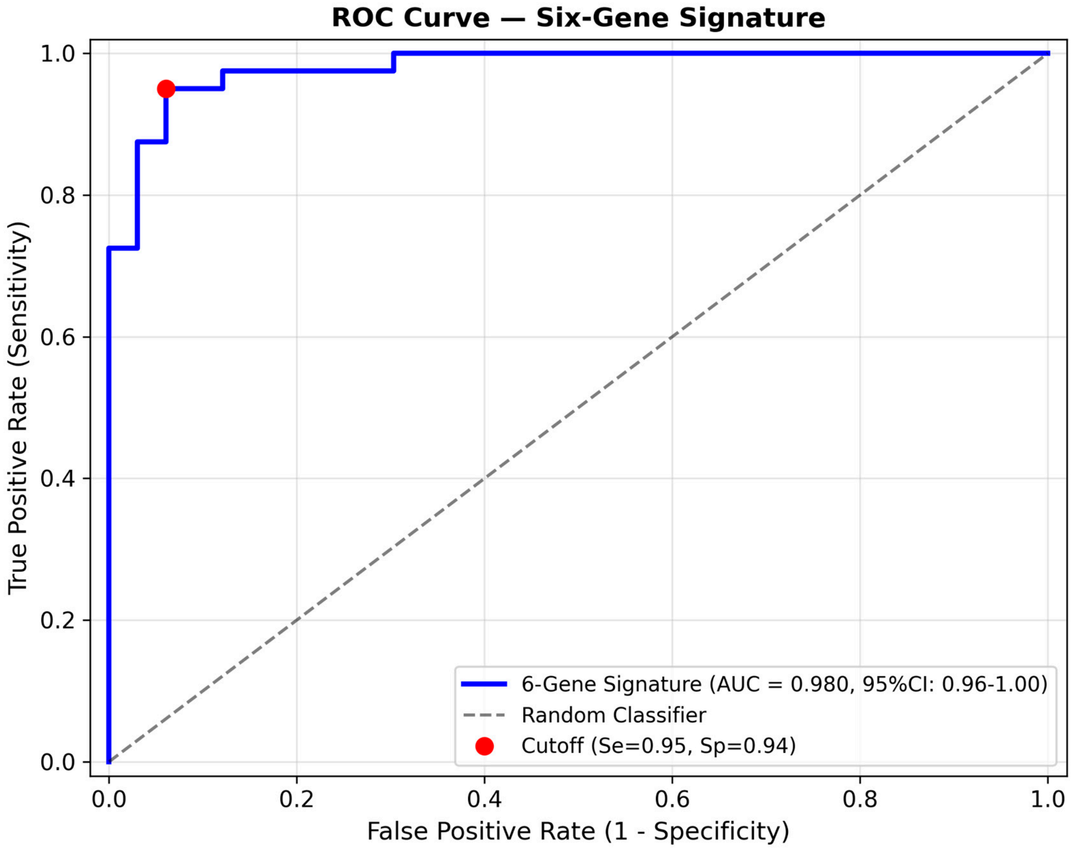

Figure S9. ROC curve for the combined six-gene diagnostic signature

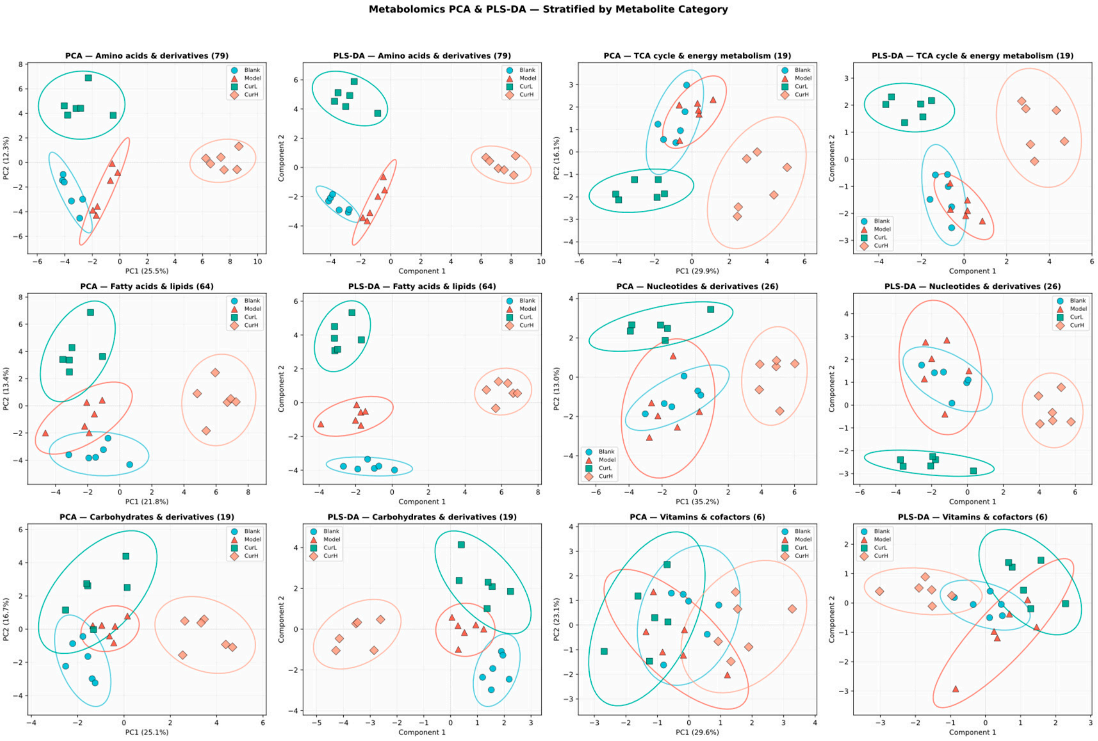

Figure S10. PCA& PLS-DA-Stratified by Metabolite Category.
